# Process evaluation of the ACTION programme: a strategy for implementing person‐centred communication in home care

**DOI:** 10.1186/s12912-021-00565-8

**Published:** 2021-04-08

**Authors:** Tanja Gustafsson, Annelie J Sundler, Elisabeth Lindberg, Pernilla Karlsson, Hanna Maurin Söderholm

**Affiliations:** 1 Faculty of Caring Science, Work Life and Social Welfare, University of Borås, Allégatan 1, SE-501 90 Borås, Sweden; 2 PreHospen Centre for Prehospital Research, University of Borås, Borås, Sweden

**Keywords:** Communication, Intervention, Implementation, Education, Home care, Nursing assistants, Older persons, Person‐centred

## Abstract

**Background:**

There is currently a strong emphasis on person-centred care (PCC) and communication; however, little research has been conducted on how to implement person-centred communication in home care settings. Therefore, the ACTION (A person-centred CommunicaTION) programme, which is a web-based education programme focusing on person-centred communication developed for nurse assistants (NAs) providing home care for older persons, was implemented. This paper reports on the process evaluation conducted with the aim to describe and evaluate the implementation of the ACTION programme.

**Methods:**

A descriptive design with a mixed method approach was used. Twenty-seven NAs from two units in Sweden were recruited, and 23 of them were offered the educational intervention. Quantitative and qualitative data were collected from multiple sources before, during and after the implementation. Quantitative data were used to analyse demographics, attendance and participation, while qualitative data were used to evaluate experiences of the implementation and contextual factors influencing the implementation.

**Results:**

The evaluation showed a high degree of NA participation in the first five education modules, and a decrease in the three remaining modules. Overall, the NAs perceived the web format to be easy to use and appreciated the flexibility and accessibility. The content was described as important. Challenges included time constraints; the heavy workload; and a lack of interaction, space and equipment to complete the programme.

**Conclusions:**

The results suggest that web-based education seems to be an appropriate strategy in home care settings; however, areas for improvement were identified. Our findings show that participants appreciated the web-based learning format in terms of accessibility and flexibility, as well as the face-to-face group discussions. The critical importance of organizational support and available resources are highlighted, such as management involvement and local facilitation. In addition, the findings report on the implementation challenges specific to the dynamic home care context.

**Trial registration:**

This intervention was implemented with nursing assistants, and the evaluation only involved nursing staff. Patients were not part of this study. According to the ICMJE, registration was not necessary ().

## Background

Today, many older persons are being cared for in their homes through the support of different types of home care services and health care professionals. In home care, there is a strong emphasis on person-centred care (PCC), even though knowledge of how to provide such care is limited. A cornerstone in PCC is communication [1–3]. Previous research has indicated a greater need for a focus on communication and that such a focus is imperative to improve the communication competency of nursing staff in home care [4]. While the importance of PCC in everyday nursing practice has been highlighted [1, 5, 6], few empirical studies have examined the implementation of *person-centred communication* in nursing practice and the home care of older persons. Therefore, the ACTION (A person-centred CommunicaTION) programme was designed to enhance communication among nursing assistants (NAs) in home care. This paper reports on the evaluation of implementation of the programme.

In Sweden, nursing and social care, e.g., help with daily living or personal care and hygiene, is performed by NAs. Most NAs in Sweden have a three-year high school education, with the option to become certified NAs through a 1.5-year nursing education programme. Structured training in communication is rarely offered to NAs. There are challenges for home care service organizations regarding how to recruit and retain nursing staff with the necessary competency, and temporary staff as well as unqualified personnel are common. Work in home care services is mobile and sometimes unpredictable. NAs move between home care units and the homes of older persons according to a combination of set schedules and unscheduled or time-critical occurrences of care needs. This poses challenges both for NAs’ required competence and the organization of work.

### Person‐centred communication in home care

PCC is widely recognized as a key concept related to the quality of nursing care and has been recognized to have a positive impact on patient care (e.g., [7, 8]) as well as on nursing staff [9–11]. Nursing activities in the care of older persons are embedded in everyday conversations, where small talk can be used to elicit information, normalize unpleasant procedures and manage interactions and relationships [12]. Understanding and showing sensitivity to older persons’ expressions are necessary for responding to individual needs and enhancing PCC [4]. The communication competency of nursing staff is a cornerstone of the achievement of nursing goals. There are, however, challenges related to communication with and care of older persons [4, 13–17], which might risk individuals being misinterpreted or ignored. Features of PCC and communication emphasize the need for skilled nursing staff, yet a relatively large proportion of employees in home care are reported to have low qualification levels, and the literature points to an imbalance between expected and actual nursing staff competency [18, 19]. NAs need support and education to develop the knowledge, skills and attitudes required to meet the individual needs of older persons [20]. Thus, research suggests that enhancement of communication may support NAs in meeting individuals’ needs.

### Implementation of person‐centred communication in home care settings

PCC has been implemented in a variety of out-of-hospital settings [21]; however, studies on implementing PCC interventions specifically in home care are scarce, with the exception of an ongoing study [22]. To this date, there are no interventions that focus on implementing person-centred*communication* in home care. Existing work has primarily focused on communication in other settings and professional groups [23–27]. Both practical efforts and research related to strategies to support NAs in becoming more person-centred in their communication are needed. Interventions in home care settings and education are complex, as several components affect and interact with each other [28], e.g., the complex practices of caring for older persons; the mobile, dynamic and often unpredictable working conditions, and challenges regarding development, delivery and evaluation of educational interventions and their effects. For competency development and educational efforts in healthcare, previous studies [29–32] have proposed flexible pedagogical approaches such as web-based or blended approaches that combine e-learning and face-to-face interactions. Such approaches have been effective for a wide range of health contexts and purposes, e.g., providing advice and support to cancer survivors [31], conducting motivational interviewing [30], and changing clinician behaviour [29]. Compared to no intervention, web-based training for health care professionals has been reported to have positive effects in terms of knowledge outcomes, skills, learner behaviour and patient effects [32]. Such findings suggest that web-based education would be an appropriate strategy for implementing person-centred communication in home care settings.

Different theoretical approaches can be used to understand and explain how and why intervention implementation does or does not work [33] and provide a systematic way to plan, implement and evaluate new interventions. This work follows the MRC guidance by Moore et al. [34] with a primary focus on evaluating the implementation strategy. Implementing person-centred communication in a home care setting is a complex pursuit, both with respect to the intervention, or *what* is to be implemented (person-centred communication), and the implementation strategy (the ACTION programme), or *how* the intervention will be implemented. The evaluation of *implementation outcomes*, which are distinct from *intervention outcomes*, could be used to understand the success (or failure) of the implementation and the process of the implementation [35]. As stated by Proctor et al., “an intervention will not be effective if it is not implemented well” ([35], p.66). The home care setting presents unique challenges for implementing interventions. To understand the outcomes of complex interventions, it is therefore crucial to start with the evaluation of implementation outcomes, i.e. conducting a process evaluation. Therefore, the aim of this process evaluation was to describe and evaluate the implementation of the ACTION programme. The following research questions are addressed:

- To what extent was the programme delivered as intended?
- How did the NAs respond to and interact with the programme?
- What contextual barriers and facilitators affected implementation?

### The ACTION programme

The ACTION programme was developed as a multifaceted implementation strategy [28, 36, 37], by combining a web-based education with short pre-recorded lectures, movie clips, reflection assignments and quizzes packaged in step-wise modules, and on-site group supervision enabling peer dialogue (Table 1). In addition, the strategy included external facilitation, local opinion leaders and computerized reminders to stimulate engagement and support programme completion.

**Table 1:** Overview of the eight educational modules

Module	Topic	Content	Pedagogical approaches
1	Introduction to PCC and communication	Introduce the educational intervention. Introduce person-centred care and communication following Carl Rogers’s [35] perspective on person-centredness.	Introductory meeting including a video lecture and a movie clip Reflective group discussion On site
2	Core values of nursing and social home care	Outline the core values of nursing and social care of older persons. Discuss ethical concerns and challenges related to communication in home care.	Video lecture Movie clip Individual reflection assignment Web-based
3	Verbal and non-verbal communication	Introduce and discuss verbal and non-verbal communication and the dimensions of power in both verbal and non-verbal communication.	Video lecture Quiz Web-based
4	Presence and active listening	Develop an understanding of the importance of engaging in active listening and being present in meetings with older persons.	Movie clip Video lecture Individual reflection assignment Web-based
5	Group supervision	Discuss and reflect on participants’ experiences of communication challenges.	Groups of 3–6 participants Led by a member of the research team Reflection exercises On site
6	End-of-life care	Discuss communication related to end-of-life care in the home care setting.	Movie clip Video lecture Individual reflection assignment Web-based
7	Person-centred and emotional conversations	Discuss how to facilitate positive interactions and person-centred communication, including in conversations about existential matters with older persons.	Movie clip Video lecture Quiz Web-based
8	Flexible and person-centred communication	Discuss how to communicate with sensitivity and be flexible to the person being cared for. Summarize the main points of the educational intervention.	Video lecture Individual reflection assignment Course leader feedback Web-based

The programme was developed by a team of researchers and teachers with expertise in the area and was tailored to the home care context. To achieve the best possible outcomes with respect to participation and acceptance, the work was guided by Everett Rogers’s [38] strategic attributes for successful implementation: to provide relative advantage compared to other strategies (e.g., traditional classroom education), to create compatibility with home care practice and work organization, and to minimize complexity (i.e., make the intervention easy to understand and access). The programme builds on following four components: (1) knowledge on key aspects of person-centred communication, (2) a self-directed and reflective learning approach, (3) flexibility and accessibility, and (4) efforts to stimulate activity. Table 2 outlines the core components and facilitating activities to support NA participation in the programme.

**Table 2:** Overview of the core components and facilitating activities of the ACTION programme

Core component	Facilitating activities
*Knowledge on key aspects of person-centred communication*	Pre-recorded lectures and movie clips. See Table 1 for module content.
*Self-directed and reflective learning*	Eight stepwise modules. Quiz and written assignments, with feedback from instructors after the last module. One group supervision session.
*Flexibility and accessibility*	Web-based education conducted on computers/tablets to give the NAs the opportunity to decide when and where to complete the educational intervention.
*Efforts to stimulate and encourage activity*	Availability of an external facilitator from the university to support the NAs to complete the programme. Weekly e-mail reminders.

#### Knowledge on key aspects of person‐centred communication

The programme content (Table 1) focused on PCC and communication in home care, and was developed based on previous research and the educational platform within the COMHOME project [39], an international research project with focus on person-centered and emotional communication between nurses and older people in home care settings. The theoretical foundation was grounded in Carl Rogers’s [40] perspective of person-centredness, which emphasizes a caring approach based on the principles and values of acceptance, caring, empathy and sensitivity in human interactions. According to this perspective, PCC involves treating patients as persons and being sensitive, respectful and compassionate, as well as supporting patients’ capacity for autonomy [5, 41]. This definition of PCC is complemented by the current literature [1–3], which adds communication, respect, autonomy, and empowerment to PCC. In addition, the work of Watzlawick et al. [42] provides a basis for understanding communication, suggesting that all interactions always involve communication and that actions, words and silences all convey a message.

The programme content was developed to provide useful knowledge compatible with the NAs daily caring encounters and was organized according to eight programme modules. As outlined in Table 1, the modules were structured around different topics of person-centred communication. For example, module 2 started with a short movie clip (from YouTube) with an older person describing experiences of aging and needing care. Thereafter, a university lecturer gave a short video talk about the core values of nursing in communication and interaction with older persons in need of care.

#### Self‐directed and reflective learning

The programme was structured in eight stepwise modules completed over eight weeks; this time frame was estimated to be feasible with respect to expected work effort and time consumption. The first four modules were pre-recorded, the fifth module consisted of live group supervision sessions at the workplace, and the last three modules were co-created with the NAs based on discussions regarding programme content and structure. A self-directed approach to learning [43, 44] was adopted to emphasize a learning process in which the NAs were supported and guided. Each module was estimated to take a maximum of 15–30 min. A final quiz or reflection assignment had to be completed in each module to proceed to the next module. The intentions for the assignments were to enhance self-directed learning, to support reflections and to prevent the participants from passively “clicking through” the pages without engaging with the content. Another strategy to enhance reflection was group supervision in the fifth module. The use of reflection is important to motivate and engage participants in person-centred practice development [45]. The assignments and group supervision were used to stimulate reflection and deep learning by using the NAs’ own experiences from caring encounters as a basis for understanding and developing their communicative ability.

#### Flexibility and accessibility

The backbone of the programme was a web-based learning platform where several types of content and learning strategies were combined (see Table 1). The web-based approach was chosen for its flexibility and accessibility, considering the NAs’ dynamic and somewhat unpredictable everyday working conditions. Access to web-based education was provided through computers located in the home care units, as well as with tablets provided by the research team. This setup allowed the NAs to decide when and where to carry out the different modules according to their workloads and schedules. Although one of the core ideas of the programme was to limit interference with the NAs’ daily work, the activities were still expected to require a certain amount of time and effort from the NAs.

When developing the programme, considerations were made to address potential differences in technical skills among the NAs. The learning platform is widely used for a variety of student groups and educational levels in Sweden, and is considered to be relatively easy to use and navigate and to require a limited amount of IT experience. Hence, some of the NAs had previous experience of using the platform. In case of technical problems, the NAs had access to support staff from the university that hosted the platform.

#### Efforts to stimulate activity

To encourage, motivate and support the NAs in completing the programme and to identify problems during the implementation process, an external facilitator (the first author) was available. As emphasized in previous research (e.g., [46, 47]), a facilitator who helps and supports participants during the implementation process is crucial. The facilitator’s interpersonal relationship with participants can make the process easier, and problems and needs for improvements can be identified and resolved early in the process [48].

Additional strategies to stimulate activity during the educational intervention included weekly e-mail reminders and paper-based newsletters to inform the NAs about the progress of the programme and encourage them to complete the current or upcoming module. The content of the e-mail reminders included a short description of what to expect from the upcoming module, which was written in an inviting and motivating way. The text of the e-mail reminders was also posted as a reminder on a bulletin board in the staff room.

## Methods

### Design

To evaluate the implementation of the ACTION programme, a descriptive evaluation design guided by Moore et al. [34] including both quantitative and qualitative data was used. The evaluation focused on the implementation of the programme and contextual factors affecting the implementation, to allow an understanding of how and why the programme did or did not work.

### Study setting and recruitment procedures

Two home care units were recruited from a middle-sized municipality in western Sweden. The inclusion criteria were home care service organizations with a manager expressing willingness to participate in and support the intervention. The intervention was performed in spring 2018. The manager decided whether NAs would be given the opportunity to participate in the programme; however, participation in the data collection was voluntary and managed by the researchers with respect to requesting participation, providing information about the study, and obtaining consent. The NAs first received information about the project during a workplace meeting approximately five months before the start of the education programme. Responsible researchers presented the overall aim of the intervention, the programme and the planned data collection.

The eligibility criterion was permanent employment (full- or part time), enabling participation throughout the programme and data collection; 27 NAs met this criterion. Of these NAs, one declined to participate in data collection, one was on long-term sick leave, and three terminated their employment. After one new employee was hired, 23 NAs were eventually offered the programme.

### Data collection

**Fig. 1:**
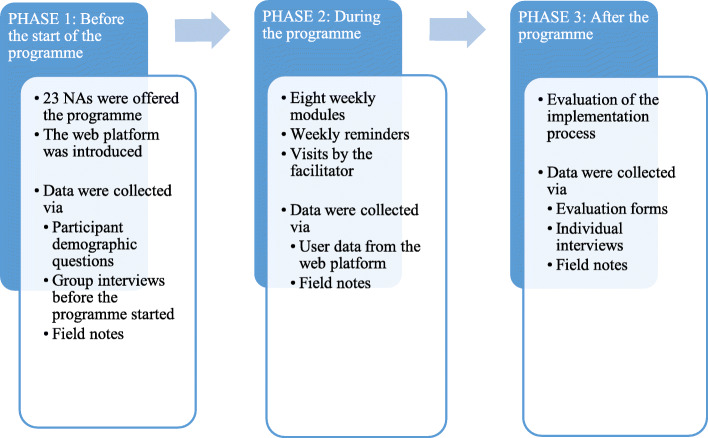
Flow chart of the implementation and data collection

In order to address the research questions, quantitative and qualitative data were collected from multiple sources during the implementation, i.e., before, during and after the programme. As suggested by Moore et al. [34], the feasibility stage of a process evaluation should combine different types of quantitative measures and in-depth qualitative data. In this study, data consisted of demographics; user analytics data; and data from pre- and post-interviews, evaluation forms, and field notes (Fig. 1). The aim of this approach was to provide a detailed basis for understanding the implementation of the programme.

*Before* the programme, data were collected via participant demographic questionnaires, group interviews and field notes. Twenty-two of the NAs participated in four group interviews, which focused on their expectations and worries about the upcoming programme, i.e., aspects that were important for tailoring the programme and its implementation. At the end of these interviews, the NAs were asked to write anonymous personal notes about their thoughts on Post-It notes collected by the researchers. *During* the programme, user data on the NAs’ participation and actions were extracted from information logs on the web-based platform. These logs included the time spent on each module, the number of visits, and the portion of each module that the participant had completed. *After* the programme, the NAs shared their experiences of the programme, regardless of how many modules they had completed, by submitting an anonymous evaluation form. The form consisted of four open-ended questions on the NAs’ experiences of the design and content of the programme. The NAs were asked to describe what they thought about the overall program format (the learning platform, lectures, movie clips and supervision) and the educational content (e.g. person-centred communication and emotional conversations). Repeated reminders to complete the form were sent by e-mail and distributed during the facilitator’s visits, and ultimately, fifteen evaluation forms were received. These responses provided a broad basis for insights into the NAs’ perceptions of the programme on a group level.Five NAs participated in individual interviews to provide in-depth descriptions of their experiences of the programme. The interviews were semi-structured and focused on aspects of participating in the education programme in terms of benefits, problems and practical matters (e.g. time and support). The interviews lasted between 30 and 60 min.

In order to capture the contextual processes and events that took place during the implementation of the programme, field notes were written by the facilitator. These included observations from visits; notes from discussions with the manager, team leaders and NAs; facilitator reflections; e-mail communications; and questions discussed among researchers.

### Data analysis

Quantitative data were analysed using IBM SPSS Statistics version 25. Descriptive statistics were used to provide demographic descriptions of the participants. User statistics from the web-based platform were used to analyse the participant flow and identify patterns in activity and use. The completion time, number of visits and assignment completion in each module were analysed. To analyse whether the program was delivered as intended, that is, whether it was feasible and accessible for NAs irrespective of demographic characteristics (e.g., language, age and work experience), t-tests and Pearson correlation tests were used.

A content analysis approach was used to analyse the qualitative data (interviews, evaluation forms and field notes) to examine the NAs’ perceptions and experiences [49]. First, the data were read several times. Meaning units were coded and grouped into descriptive categories. In the results, quotations from the interviews and evaluation forms were used to illustrate the NAs’ experiences. The analysis was mainly performed by the first and last authors and was further discussed and validated by all authors. All authors read and commented on the final version of the analysis to ensure the rigour of the categories described.

## Results

The results are presented in three main sections: (I) demographic characteristics of the participants; (II) implementation process in terms of the NAs’ programme attendance and activity and their experiences of the programme format and learning approach; and (III) contextual enablers and barriers, including external and local facilitators and challenges related to time, place, equipment and work load.

### I. Demographic characteristics of the participants

The demographic characteristics of the participating NAs (n = 23) are presented in Table 3. The median age and gender distribution of the participants were in line with the current overall characteristics of NAs, as the NA profession is female dominated. The NAs’ years of experience working in home care varied. One-third (n = 7) of the participants had a native language other than Swedish.

**Table 3:** Demographic characteristics of the participants (*n* = 23)

Characteristics	
Age, years, median (range)	39 (24-61)
Gender, female/male (n)	20/3
Employment, full-time (100%)/part-time (60-90%)/unknown (n)	5/13/5
Home care work experience, years, median **(**range)	7 (1-40)
Years at the unit, median **(**range)	4 (0-18)
Certified NAs, yes/no (n)	21/2
Native language, Swedish/other (n)	16/7

### II. Implementation process

The evaluation of the NAs’ attendance and activity in the ACTION programme showed higher participation in the first five modules, followed by declining activity in the remaining modules. The interviews revealed both positive and negative experiences with respect to the programme format and pedagogical approach.

#### Programme attendance and activity

The majority of the NAs participated in the education programme: 91 % (*n* = 21) completely or partially participated in at least five of the eight modules. Twenty-one NAs also participated in the on-site group supervision (the fifth module). After this module, a gradual decrease in attendance occurred. The last module, and thus, the full education programme, was completed by half of the NAs (*n* = 12, 52 %). Details on the participation rates in the modules and assignments are presented in Table 4.

**Table 4:** Participation rates in the modules and assignments

Module	Content	Participation rate (n) ^a^
**Module 1**	Introduction (on-site)	20
**Module 2**	Movie clip	21
	Video lecture	21
	Reflection assignment	22
**Module 3**	Video lecture	20
	Quiz	22
**Module 4**	Movie clip	22
	Video lecture	19
	Reflection assignment	21
**Module 5**	Group supervision (on-site)	21
**Module 6**	Movie clip	18
	Video lecture	15
	Reflection assignment	16
**Module 7**	Movie clip	14
	Video lecture	13
	Quiz	15
**Module 8**	Video lecture	12
	Reflection assignment	11

^a^Participants who spent only a few seconds in the modules were excluded

The activity data (number of visits to sub-pages, time spent on modules and sub-pages, and activity completion) indicated that the participants tended to interrupt or skip parts of the modules that had longer lectures. Quizzes and reflective assignments needed to be completed to proceed to the next module, but there did not seem to be any differences in completion rates based on whether the module included a quiz or written reflection assignment.

No correlations were found between attendance and variables such as age, work experience, or workplace tenure, and there were no gender differences. There was, however, a significant difference (*p = .005*) in the number of visits to each page between native and non-native Swedish speakers. Non-native speakers showed more than twice the amount of activity in terms of the number of visits to the sub-pages in the modules and spent more time on each module than participants who were native Swedish speakers. Nevertheless, all of these participants completed the entire education programme.

#### Experiences of the programme format

Overall, the NAs’ expectations of the programme included both curiosity and concern, as expressed in the first round of interviews. Not knowing what was to come was exciting and led to the NAs’ interest in how the content could contribute to their knowledge and competence development.

“It’s still exciting to see what this leads to and what one can gain from it.” (Group interview 3).

The education started with an on-site introductory meeting, where all participating NAs were introduced to the web platform and instructed on how to use it. They also participated in the first module, which included a pre-recorded lecture and a short movie clip. The introductory meeting ended with group discussion, during which the NAs reflected on the module content. During this meeting, there were also opportunities to ask questions regarding practical matters such as the web platform or other issues. After this first meeting, all following modules were conducted individually, except the fifth (the on-site group supervision).

The NAs expressed that compared to traditional education, the web-based education format had benefits such as accessibility and flexibility for the NAs to independently decide when to complete the modules. The tablets and headphones offered were appreciated by the NAs, as they allowed for additional flexibility and privacy.

“I think that it [the education programme] was great! It was great that you could log in and then that it was stepwise. // It gives you more independence. I think that it was very positive!” (Individual interview 4).

“I have done them [the modules] on the tablet. Very good! // I just needed to sit down for a while and shut everything out. It was great!” (Individual interview 4).

Overall, the NAs described that the web-based education format to be easy to use and appreciated the stepwise modules. Some NAs found the web format to be challenging due to a lack of digital experience.

“Too much using the computer, especially when you’re not that technical.” (Evaluation form 3).

The web-based format allowed the research team to follow the NAs’ progression through the education programme. Consequently, the *format* allowed the early detection of NAs who were falling behind or did not follow the modules as instructed. These NAs were contacted through e-mail or during the visits by the external facilitator, and they were offered help in solving technical or other problems.

The NAs reported that the content was relevant and interesting and that it provided valuable knowledge that would be useful in their daily work. NAs with extensive work experience in home care settings expressed already having sufficient knowledge. They felt that parts of the content were repetitive, but they valued the opportunity to reflect on their everyday work practices. Some NAs also believed that the intervention was more suitable for NAs who did not have previous work experience.

“Yes, I think it [the intervention] is useful. It actually relates to what we do all the time at work. Very useful. /…/ So, it was needed. Even if you already have some awareness of it, it’s always needed … to get more knowledge or to think about it even more.” (Individual interview 3).

“I think that the education was great, above all, for those with limited experience in healthcare, but not that many new insights for me, as I have been working for more than twenty years.” (Individual interview 2).

#### Experiences of the pedagogical approach

The NAs reported that the quizzes were enjoyable and that the reflection assignments were meaningful parts of their learning but that they were time consuming. Participants completing the reflection assignment in the final module received written feedback from the researchers. This feedback was reported to be valuable since it provided confirmation on how the NAs reflected on their current communication ability.

“I think that the reflective assignments give you more… They feel better… but they are more burdensome and time consuming.” (Individual interview 3).

The short movie clips were described as particularly interesting and stimulating. These clips provided nuance and deepened the NAs’ understanding.

“Some of the movie clips have been good. I remember one in particular that was about end-of-life care. The nurse assistants in the movie did it all so beautifully, and it consisted of the most important parts.” (Evaluation form 9).

The NAs described that the overall structure of and variation in the content were appreciated, but some NAs expressed that the education programme consisted of too many modules.

“I think that the education was lengthy, too many modules. Maybe it would have been more interesting if it was shortened a bit.” (Evaluation form 9).

Some of the lectures were perceived as too long, resulting in a loss of interest. When possible, this feedback was taken into account; for example, in the development of the later modules of the programme, shorter lectures and more engaging approaches, such as conversation-based lectures including several presenters, were used.

Another weakness with the self-directed and web-based approach was the lack of interaction with lecturers. The discussion during the group supervision were reported to be one of the most appreciated parts of the programme. It allowed NAs to reflect with colleagues on different aspects of communication. The content became more meaningful and interesting.

“It was great, the group session. It was interesting. It gave me a lot. Yes. I think everybody thought so. It was great; that is something we said afterwards.” (Individual interview 2).

The opportunity to discuss difficult matters with colleagues was described as valuable and provided the NAs with useful insights into their daily work. The NAs expressed a desire for more group sessions to meet with colleagues and to reflect on different aspects of the NAs’ everyday practice. Others thought that one session was sufficient.

### III. Contextual enablers and barriers

Contextual enablers and barriers were identified during the evaluation of the implementation, including external and local facilitators and challenges with time, space and equipment needed to participate in the education. Workload and motivation were identified as additional contextual factors crucial for the implementation process.

#### External and local facilitators

Throughout the intervention, the external facilitator acted as the primary contact between the NAs and the project team. This facilitator regularly visited the home care units approximately once a week. Another member of the research team also performed repeated visits to support and evaluate the implementation. The duration of the visits were usually 1–2 h. In addition, all NAs, team leaders and managers had access to facilitator support via phone or email. The use of this support was infrequent and mostly used for technical support questions.

At both of the home care units, team leaders had the main responsibility for organizing and planning the NAs’ daily work and schedules and dealing with changes and rescheduling when acute care situations occurred. These team leaders also became the external facilitator’s primary contact with the organization and NAs. They assisted in passing on information to the NAs, e.g., by frequently providing reminders to the NAs during the home care unit regular meetings. Although not designated or identified beforehand, their role gradually developed into that of an informal local facilitator and became a part of their work. An interpersonal relationship gradually developed between the external facilitator and the team leaders. This relationship facilitated the implementation of the intervention, established trust and enabled the facilitator to obtain an “inside” perspective of the everyday work at home care units. During on-site visits from the facilitator, team leaders expressed that they appreciated the support and positive energy from the facilitator, which helped them manage the additional work generated by the project.

#### Challenges in finding time

Early in the process, some NAs expressed concern regarding the potential time constraints of completing the education modules and the management of the required time during busy working hours.

“How would we get the time needed for this? That is something to think about. But, if this is something we will do, then time needs to be allocated, I guess. Yes. But I don’t know how.” (Group interview 4).

As anticipated, problems with time constraints occurred during the first weeks. Although NAs described that they appreciated the flexibility of the programme, a high level of responsibility was required from the NAs to balance the programme with their work duties and time management constraints.

“The most positive thing about the web platform is that it is always available. The idea that you can take advantage of vacant hours at work is good, but it requires a great deal of individual responsibility.” (Evaluation form 6).

The research team planned the programme structure and format to support the NAs’ participation, but the managers and team leaders were responsible for organizing the time and resources needed for the programme within the units. The problem with time was discussed during a workplace meeting a few weeks into the programme, and additional time was allotted in the NAs’ schedules. Nevertheless, the time assigned to participate in the programme competed with other administrative tasks, making it challenging for the NAs to complete each module on time, particularly during periods of high workload. To enable the NAs to catch up, module deadlines were extended twice during the programme, prolonging the education from eight to ten weeks.

#### Challenges related to location and equipment

Another challenge noted in the early stage of the implementation was the limited number of desktop computers, with two per home care unit allocated for the intervention and the NAs’ everyday work. Some NAs expressed worries concerning noise and privacy when they listened to the web-based lectures and completed the writing assignments. To ensure appropriate resource allocation, enable privacy to engage with the module content, and establish a sense of empowerment, the researchers decided to provide the units with tablets and headphones. Nevertheless, issues arose regarding the physical location where the NAs were expected to work with the course material. The manager’s recommendation was to primarily complete the programme at the workplace during work hours. If necessary, e.g., due to a tight schedule or heavy workload, the completion of the modules from home was allowed and compensated for as over-time. To not be disturbed or interrupted by colleagues or acute patient situations, some NAs preferred to complete the education programme at home.

#### Workload and motivation

The ACTION programme was implemented during the spring and finished approximately one month before the start of the first summer vacation period. During this time, the NAs and team leaders expressed having an accelerating workload caused by several absences due to sick leave combined with problems accessing extra staff. This situation was particularly pressing for team leaders, who were responsible for scheduling. In addition, some of the team leaders terminated their employment during the intervention implementation and were replaced by other NAs at the home care units.

Among the NAs, there was a desire to engage in the modules and a clear positive attitude towards the programme. However, some NAs perceived the intervention as burdensome, and a few expressed that they felt forced to do the programme. Some expressed frustration with the lack of involvement in the decision phase. The education programme imposed an additional workload that influenced their motivation to complete the modules. Other aspects that reduced their motivation during the intervention were vacations, sick leaves, and other prioritized training.

“This [the intervention] is something that the manager has agreed to, and then we have to accept that. End of discussion.” (Individual interview 4).

## Discussion

Overall, the programme was delivered as intended apart from some adaptions to the implementation strategy. The adaptations included a two-week programme extension, modification of the last lectures, provision of tablets, and allocation of time for the education programme in the NAs’ schedules. These adaptations were necessary to support the NAs in completing the education programme and to facilitate its implementation. The core programme components remained unchanged, which can be regarded as a strength of the implementation strategy.

It is challenging to develop content that is suitable for learners when there are variations in the participants’ work experience, age and education. The NAs perceived the content of the programme to be important and relevant; however, some of the more experienced NAs expressed that the *knowledge on key aspects of person-centred communication* (first core component) was too basic for them. Acceptability, in terms of NAs’ satisfaction with the programme content and its usefulness in practice, needs to be supported because of its interrelations with other implementation outcomes [35]. Therefore, if participants do not find the content to be relevant in practice, i.e. if the content has low relevance, this can have negative effects on outcomes related to adoption and penetration. The interviews revealed that the NAs perceived both the programme and some of the lectures to be too long, which also reduced the acceptability. The decline in participation in the last three modules might be explained by exhaustion and loss of motivation. A shorter duration and more focused programmes and modules may be preferable. Another challenge with the programme was making the content interesting to the NAs, which is an important factor to increase motivation [43, 44]. One-way communication risks NAs becoming passive receivers. To prevent this, group supervision was included in the programme with the aim of supporting *reflective learning*. The interviews emphasized the need for the NAs to gather with their peers and discuss caring activities, which was potentially underestimated in designing the programme. An additional group session would have been sufficient.

The *self-directed learning approach* [43, 44] and the programme’s *flexibility and accessibility* [29–32] demanded a high level of participant involvement, motivation and responsibility. Both the physical environment, e.g., the equipment and location, and the workplace culture need to be supportive. Such facilitating conditions have been identified as a crucial factor for the successful implementation of new technologies and tools in organizations [38, 50]. The addition of resources such as headphones and tablets was appreciated by the NAs. Although it did address the issues related to physical space by providing quiet for the NAs to concentrate on the educational content, it did not compensate for the lack of time that the NAs had to engage with the course content. The NAs were expected to complete the education programme when they had time during ordinary workdays. This approach failed almost immediately. The NAs were frustrated when they did not have the time they needed, which indicates that they were motivated and willing to undergo the education. This finding also indicates the important role of management attitudes and commitment, which is in line with previous research [50] emphasizing the importance of managerial efforts both pre- and post-implementation. Commitment may be required from all levels to support active learning and achieve a potential culture change.

The external facilitator from the university aimed to *stimulate and encourage NA activity*. Throughout the process, there were challenges in gaining access to the NAs. Although the manager initially facilitated access to the NAs, it was difficult due to the NAs’ work conditions. During the facilitator’s on-site visits, contacts were primarily made with the team leaders and occasionally with the manager but seldom with the NAs. Most of the information and support offered by the facilitator was addressed to the team leaders, making them informal local facilitators who became crucial in supporting the implementation and participant involvement. Local facilitator(s) should be assigned in the planning phase rather than emerging during the implementation process, and the different roles of those involved, as well as the functions and expectations related to these roles, should be identified, planned for and clarified, as emphasized by previous research [38, 51].

In implementation research, there is an ongoing discussion about which strategies should be used, yet evidence is scarce due to a lack of systematic reviews. Strategies may work differently according to local circumstances, which makes it difficult to create general recommendations. As this study shows, home care settings and NAs’ work constitute a dynamic and ever-changing context for implementing interventions. Moore et al. [34] state that “even where an intervention itself is relatively simple, its interaction with its context may still be highly complex” (p.2). The implementation process is multidimensional and dynamic and places high demands on researchers’ ability to develop implementation strategies that consider the specifics of the intervention, the setting, and the scientific rigor. Hence, implementation success (or failure) is related to several factors, and the strategies used and local circumstances are central; thus, there is a need for more research that helps guide researchers down the winding roads of implementation in healthcare.

### Study strengths and limitations

Conducting, implementing and evaluating complex interventions are challenging [28]. This small-scale study attempted to address some of these challenges in home care. Although an analysis of intervention outcomes (person-centred communication) was not included in this study, the findings show some promise with respect to how this type of intervention can be implemented. Nevertheless, a number of limitations need to be recognized, including the number of included participants, interpretation of the data, and involvement of researchers during the process.

The number of participants and the specifics of the local context may limit the study generalizability and impact, as in any small-scale intervention. Nonetheless, as recommended by the MRC guidelines [28], studies similar to the current study are important since they examine key uncertainties that are identified during intervention development and implementation. The experiences reported in this study contribute knowledge of factors that appear to influence the applicability and use of this type of implementation strategy.

In this study, we chose a mixed-method approach. Both qualitative and quantitative approaches have their benefits and limitations with respect to data collection and interpretation. The interpretation of the data from the web-based platform should be performed with care (as in any indirect observation); for example, it is impossible to determine the exact reasons for the amount of time spent or actions taken on a particular course module page. As such, the post-interviews provided valuable complementary insights. However, there were some potential limitations regarding the data collection. Throughout the intervention, the first author had the main responsibility for the implementation and evaluation processes (e.g., completion of the evaluation forms and interviews) and was responsible for developing some of the course modules. This involvement generated both benefits, e.g., establishing trust, and risks, e.g., influencing whether participants felt that they could be honest during interviews. To address this issue, the evaluation also included anonymous written individual evaluation forms. As reported, these forms included both positive and negative feedback.

## Conclusions

To our knowledge, this is the first study to use a web-based approach to implement person-centred communication among NAs in home care. The evaluation of the programme identified several factors that affected the process that need to be considered in future interventions in the home care context. Web-based education seems to be an appropriate strategy in this context; however, areas for improvement were identified. Our findings show that participants appreciated the web-based learning format in terms of accessibility and flexibility, as well as the face-to-face group discussions. Hence, in future delivery modes of the ACTION programme, it is important to consider the balance between different learning components. The critical importance of organizational support and available resources are highlighted, such as management involvement and local facilitation. In addition, the findings report on the implementation challenges specific to the dynamic home care context.

## Data Availability

The dataset generated and analysed during the current study is available from the corresponding author on reasonable request.

## Abbreviations

PCC: Person-centred care
NA: Nursing assistant
